# Improvement of the Airflow Energy Harvester Based on the New Diamagnetic Levitation Structure

**DOI:** 10.3390/mi14071374

**Published:** 2023-07-04

**Authors:** Long Zhang, Hang Shao, Jiaxiang Zhang, Deping Liu, Kean C. Aw, Yufeng Su

**Affiliations:** 1 School of Mechanical and Power Engineering, Zhengzhou University, Zhengzhou 450001, China; gffrfzl@126.com (L.Z.); 202022202013965@gs.zzu.edu.cn (H.S.); 15038554491@163.com (J.Z.); ldp@zzu.edu.cn (D.L.); 2 Department of Mechanical and Mechatronics Engineering, University of Auckland, Auckland 1010, New Zealand; k.aw@auckland.ac.nz

**Keywords:** push–pull diamagnetic structure, energy harvester, energy conversion rate, electromagnetic

## Abstract

This paper presents an improved solution for the airflow energy harvester based on the push–pull diamagnetic levitation structure. A four-notch rotor is adopted to eliminate the offset of the floating rotor and substantially increase the energy conversion rate. The new rotor is a centrally symmetrical-shaped magnet, which ensures that it is not subjected to cyclically varying unbalanced radial forces, thus avoiding the rotor’s offset. Considering the output voltage and power of several types of rotors, the four-notch rotor was found to be optimal. Furthermore, with the four-notch rotor, the overall average increase in axial magnetic spring stiffness is 9.666% and the average increase in maximum monostable levitation space is 1.67%, but the horizontal recovery force is reduced by 3.97%. The experimental results show that at an airflow rate of 3000 sccm, the peak voltage and rotation speed of the four-notch rotor are 2.709 V and 21,367 rpm, respectively, which are 40.80% and 5.99% higher compared to the three-notch rotor. The experimental results were consistent with the analytical simulation. Based on the improvement, the energy conversion factor of the airflow energy harvester increased to 0.127 mV/rpm, the output power increased to 138.47 mW and the energy conversion rate increased to 58.14%, while the trend of the levitation characteristics also matched the simulation results. In summary, the solution proposed in this paper significantly improves the performance of the airflow energy harvester.

## 1. Introduction

With the rapid development of wireless sensor networks [1], microelectromechanical systems [2], and the Internet of Things [3], more and more demands are being placed on portable power supplies. Traditional chemical batteries cannot provide a long-term and stable power supply [4] due to their low energy density, inability to be recycled, and environmental hazards [5]. The collection of energy from the environment and its conversion into electrical energy for use in electronic devices has been the subject of much research to meet the power requirements of these devices [6]. Energy harvesting technologies involve conversion mechanisms such as electromagnetic [7,8], triboelectric [9,10], electrostatic [11,12], magnetostrictive [13,14], thermoelectric [15,16], piezoelectric [17,18] and photovoltaic [19,20]. Energy harvesters typically use one or more conversion mechanisms to convert energy from nature: tides, vibrations, air currents, heat, etc., into electrical energy.

Airflow is a kind of widespread source of clean energy in nature, and it has many sources and the widest range of applications. Xin et al. [21] propose a two-dimensional airflow energy harvester that collects vibrational energy from the airflow in all directions via cantilever beams and piezoelectric tubes, achieving an output power of 0.353–0.495 mW at wind speeds of 8 m/s. Wang et al. [22] proposed a non-contact piezoelectric wind energy harvesting device to harvest wind-excited vibration energy with an adjustable structure to suit different wind speeds, with a maximum output of 1.438 mW at a wind speed of 40 m/s. Wang et al. [23] proposed an improved method based on a flapping airflow energy harvester, introducing flexible wing sections to increase the output power, which reached about 930 mW at a wind speed of 9 m/s when the flexible section was 5 cm. In all these articles, the output performance of the airflow energy harvesters is not high, while the gap between the collected energy and the electrical energy output is too large to achieve a high energy conversion rate.

Diamagnetic levitation was first investigated experimentally by Cansiz and Hull [24] in 2004. The non-contact nature of the diamagnetic levitation structure avoids friction between moving parts, so it can be used in airflow energy harvesters to achieve high output performance. In our previous work [25], a push–pull diamagnetic levitation structure was reported, and the new diamagnetic levitation structure was used to harvest airflow energy. The airflow energy harvester [26] operated with good stability and environmental adaptability.

This paper focuses on improving the airflow energy harvester based on the new diamagnetic levitation structure to enhance output performance and energy conversion rate. A centrosymmetric rotor is proposed to address the shortcomings of the three-notch rotor. Simulation models are built in COMSOL to compare and verify various floating rotors’ output performance and determine the optimum rotor parameters. A joint COMSOL and MATLAB simulation was carried out to compare the levitation characteristics of the new rotor with those of the three-notch rotor. Experimental prototypes and platforms have been built based on theoretical and simulated models, and the results show that the airflow energy harvester with the new rotor has higher output performance and better levitation characteristics.

## 2. Theoretical Analysis

### 2.1. Analysis of Fundamentals

**Figure 1:**
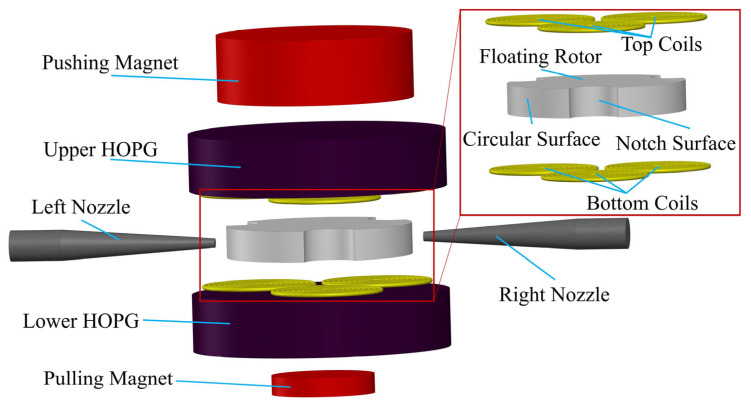
3D schematic of the airflow energy harvester.

The three-dimensional model of the airflow energy harvester is shown in Figure 1. It includes the pushing magnet, the upper highly oriented pyrolytic graphite (HOPG) sheet, top coils, the floating rotor, bottom coils, the lower highly oriented pyrolytic graphite sheet, the pulling magnet, and two airflow nozzles. All the structures except the nozzles are arranged coaxially. The two nozzles are placed symmetrically around the vertical axis of the energy harvester and located in the central horizontal plane of the floating rotor.

**Figure 2:**
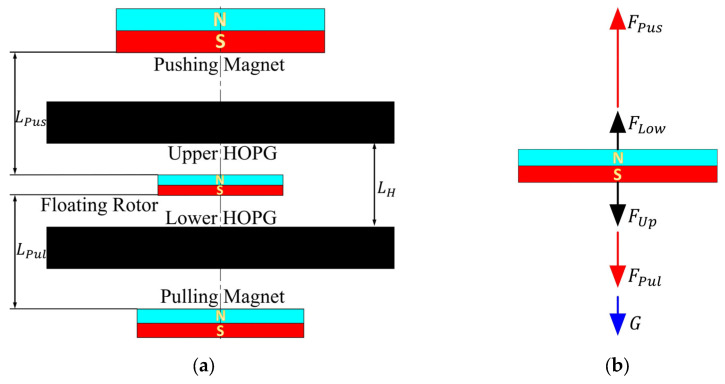
Schematic diagram of the new diamagnetic levitation structure: (**a**) structure schematic diagram; (**b**) force diagram of the floating rotor.

The push–pull diamagnetic levitation structure is shown in Figure 2a, the floating rotor has the same magnetization direction as the pushing magnet and pulling magnet, so the floating rotor is subject to their magnetic attraction ***F_Pus_*** and ***F_Pul_***. Diamagnetic force ***F_Up_*** and ***F_Low_*** are exerted on the floating rotor from the upper and lower HOPG sheets. In Figure 2b, the axial resultant force of the floating rotor can be expressed as,

(1) $$F_{R}=F_{Pus}+F_{Low}-F_{Pul}-G-F_{Up}$$

where ***G*** represents the gravity of the floating rotor, and ***F_R_*** denotes the axial resultant force. The potential energy of the floating rotor can be expressed by Equation (2).

(2) $$U=-\vec{M}\cdot \vec{B}+mgz=-\vec{M}\left(\vec{B_{Pus}}-\vec{B_{Pul}}\right)+mgz=-M\left(B_{Pus}-B_{Pul}\right)+mgz$$

where ***M*** is the magnetic dipole moment of the floating rotor, ***B_Pus_*** denotes the magnetic flux density of the pushing magnet, ***B_Pul_*** shows the magnetic flux density of the pulling magnet, *m* shows the mass of the floating rotor, and *z* shows the distance from the ground. Equation (3) can be obtained by substituting the diamagnetic influence exponents [27] *C_z_* and *C_r_* into Equation (2).

(3) $$U=-M\left\{\begin{array}{c} \left(B_{Pus0}-B_{Pul0}\right)+\left[{\left(B_{Pus}-B_{pul}\right)}^{\prime }-\frac{mg}{M}\right]z+\frac{1}{2}{\left(B_{Pus}-B_{Pul}\right)}^{\prime \prime }z^{2}\\ +\frac{1}{4}\left[\frac{{{\left(B_{L}-B_{P}\right)}^{\prime }}^{2}}{2\left(B_{L0}-B_{P0}\right)}-{\left(B_{Pus}-B_{Pul}\right)}^{\prime \prime }\right]r^{2}+\cdots \end{array}\right\}+C_{z}z^{2}+C_{r}r^{2}$$

The formula for the stability of the floating rotor that can be derived from Equations (3)–(5) are the equations for vertical and horizontal stability.

(4) $$K_{v}\equiv C_{z}-\frac{1}{2}M{\left(B_{Pus}-B_{Pul}\right)}^{\prime \prime }>0\;(\mathrm{Vertical}\;\mathrm{stability})$$

(5) $$K_{h}\equiv C_{r}+\frac{1}{4}M\{{\left(B_{Pus}-B_{Pul}\right)}^{\prime \prime }-\frac{m^{2}g^{2}}{2M^{2}\left(B_{L0}-B_{P0}\right)}\}>0\;(\mathrm{Vertical}\;\mathrm{stability})$$

Compared with the old structure [28], the push–pull diamagnetic levitation structure only introduces a pulling magnet, but has the advantages of multiple levitation equilibrium points, multiple maximum monostable levitation spaces, a large increase in horizontal recovery force, and more stable axial force distribution. The airflow energy harvester with the push–pull diamagnetic levitation structure has better output performance and a wider application range.

### 2.2. Analysis of the Floating Rotor

**Figure 3:**
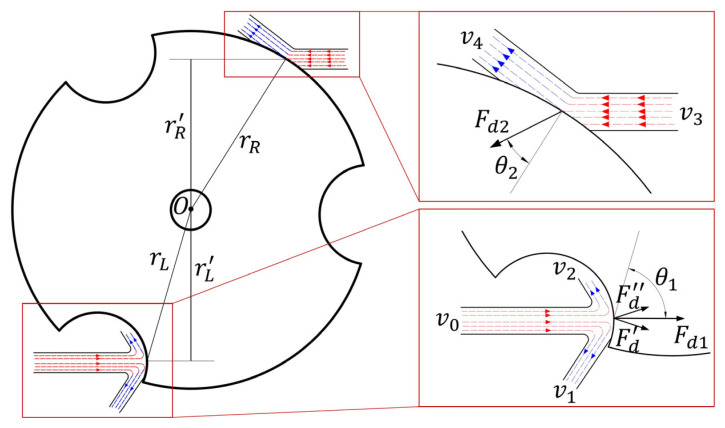
Diagram of the driving forces on the floating rotor.

The schematic diagram of the three-notch rotor is shown in Figure 3, with its radius, thickness, notch radius and central hole radius being 9 mm, 3 mm, 2.5 mm, and 1 mm, respectively. The floating rotor rotates around the central axis under the driving of two symmetrical and nozzle airflow. When the airflow collides with the floating rotor, the motion state of the airflow will be changed. According to the momentum theorem, the force between the floating rotor and the airflow is generated, which changes the velocity direction and magnitude of the airflow, and the floating rotor starts to rotate under the action of this force.

When the airflow collides with the floating rotor, the motion state of the airflow can be simplified as shown in Figure 3, where red represents the airflow before the collision with the rotor and blue represents the airflow after the collision with the rotor. The driving forces at the notch surface and the circular surface are ***F_d_*_1_** and ***F_d_*_2_**, respectively.

(6) $$F_{d1}=F_{d}^{\prime }+F_{d}^{\prime \prime }={∭}_{V_{1}}\rho v_{1}dV-{∭}_{V_{1}}\rho v_{0}dV+{∭}_{V_{2}}\rho v_{2}dV-{∭}_{V_{2}}\rho v_{0}dV$$

(7) $$F_{d2}={∭}_{V}\rho v_{4}dV-{∭}_{V}\rho v_{3}dV$$

Connecting the collision point of the airflow and the center of the floating rotor, *θ* represents the angle between the driving force and the line connecting the two. When *θ* is closer to 90°, the driving effect on the rotor is more prominent, while the radial force on the rotor is smaller. It can be seen from Figure 3 that the angle *θ*_1_ is greater than *θ*_2_, and the tangential component of the driving force ***F_d_*_1_** is larger, while the radial component of the driving force ***F_d_*_2_** is larger.

**Figure 4:**
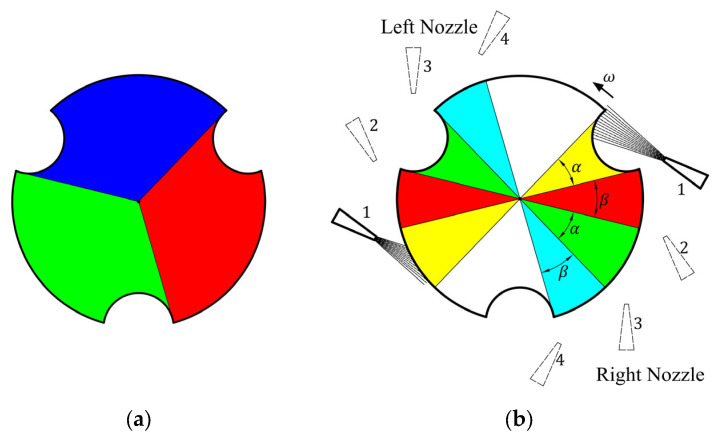
The floating rotor area division: (**a**) rotation period division; (**b**) motion state period division.

According to the relative positions of the nozzle and itself, the floating rotor can be divided into three parts, red, green, and blue areas shown in Figure 4a, each of which is 120°. In a whole rotation of the floating rotor, the airflow from the right-side nozzle will blow to three areas in turn. Therefore, the motion state of the floating rotor will undergo three periodic changes in each rotation. As shown in Figure 4b, each period can be divided into four stages according to the different positions of the left and right-side nozzles. During the four stages of each period, the relative position between the floating rotor and the nozzles changes sequentially from position 1 to position 4, while the airflow is directed towards the floating rotor’s yellow, red, green, and cyan areas, respectively. Furthermore, the angle *α* of the yellow and green areas is 32°, and the angle *β* of the red and cyan areas is 28°.

(8) $$F_{H}=F_{r}=F_{Ldr}+F_{Rdr}\;$$

**Figure 5:**
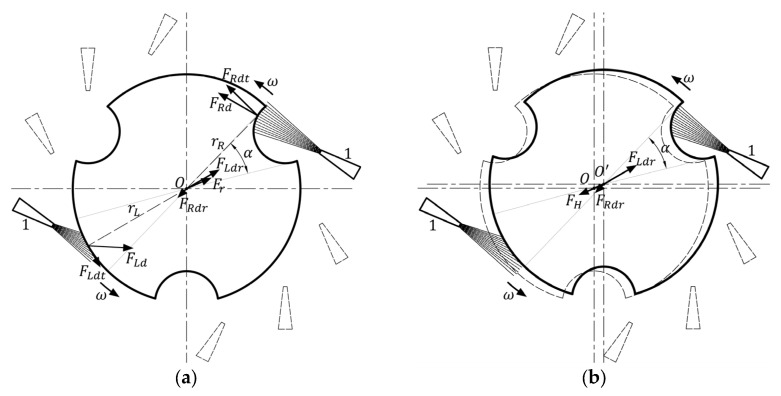
First stage: (**a**) the floating rotor at point *O*; (**b**) the floating rotor at point *O*’.

**Figure 6:**
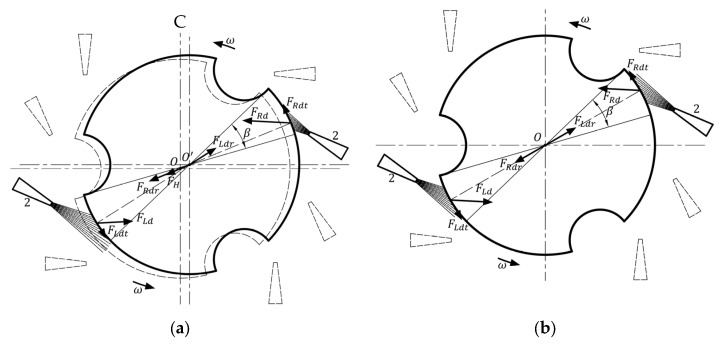
Second stage: (**a**) the floating rotor at point *O*’; (**b**) the floating rotor at point *O*.

The first stage is shown in Figure 5. In Figure 5a, the driving force of the airflow from the right side and left nozzles on the floating rotor are ***F_Rd_*** and ***F_Ld_***, respectively. The two forces are decomposed into two tangential forces ***F_Rdt_*** and ***F_Ldt_***, and radial forces ***F_Rdr_*** and ***F_Ldr_***. As can be seen from Figure 3, the two radial forces cannot completely cancel each other out; hence, the resultant force of the two is ***F_r_***, which makes the floating rotor unbalanced in the horizontal plane and thus begins to offset. When the pushing magnet, the pulling magnet, and the floating rotor are not coaxial, the pushing magnet and pulling magnet exert a horizontal magnetic force on the floating rotor, and the sum of the two horizontal magnetic forces is the horizontal recovery force ***F_H_*** of the floating rotor. In Figure 5b, the floating rotor begins to offset under the action of radial force, and with the increase in the offset, the horizontal recovery force ***F_H_*** also increases. As expressed in Equation (8), when it reaches *O’*, the value of the horizontal recovery force ***F_H_*** is equal to that of the radial force ***F_r_***, the floating rotor reaches its equilibrium in the horizontal direction, and the offset also reaches the maximum value. *O*’ is denoted as the maximum right side offset point of the floating rotor, where the floating rotor continues to rotate and enters the next stage, as shown in Figure 6.

(9) $$F_{Ldr}=F_{Rdr}\;$$

In Figure 6a, at the beginning of the second stage, since the floating rotor is still located at the maximum right-side offset point, the right-side nozzle is closer to the floating rotor, so the right-side driving force ***F_Rd_*** is significantly greater than the left driving force ***F_Ld_***. In the horizontal direction, the floating rotor will return to the original central *O* point under the combined action of the horizontal recovery force ***F_H_***, the right-side radial force ***F_Rdr_*** and the left radial force ***F_Ldr_***. In Figure 6b, when the floating rotor returns to point *O*, the left and right-side nozzles are at an equal distance from the floating rotor, the radial and tangential components of the left and right-side driving forces ***F_Ld_*** and ***F_Rd_*** are equal, as shown in Equation (9). The floating rotor enters the third stage after 30° rotation around *O*.

In the third stage, the airflow from the left nozzle blows toward the notch surface, while the right nozzle blows toward the circular surface, which corresponds to the rotation of the floating rotor by 180° relative to the first stage. At this time, the direction of the horizontal resultant force ***F_r_*** is exactly opposite to the horizontal resultant force in the first stage, the floating rotor will be offset to the left, finally reaching the maximum left offset point *O*”. The floating rotor will enter the fourth stage with another 30° rotation. The fourth stage is like the second one in that the floating rotor returns to the initial point *O* again under the action of the horizontal recovery force ***F_H_*** and the two radial forces. The entire period is the completion of the rotation of the floating rotor before entering the next rotation period.

**Figure 7:**
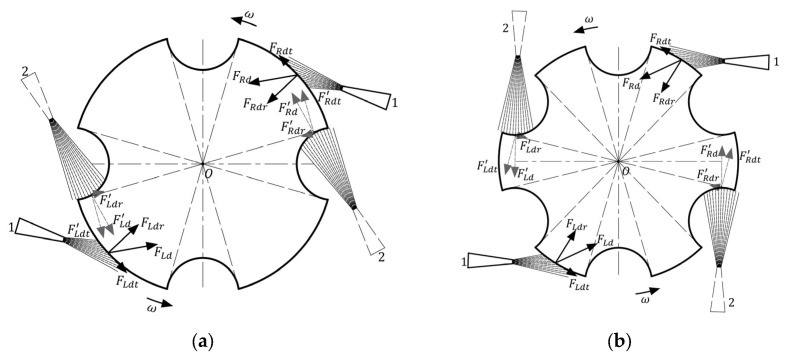
The centrosymmetric rotors: (**a**) the four-notch rotor; (**b**) the six-notch rotor.

During one rotation cycle, the floating rotor is offset to the left and right in the horizontal plane in turn because radial forces cannot be fully counteracted. After reaching a maximum speed of 20,000 rpm, the floating rotor undergoes more than 900 periodic offsets per second. Such a high frequency of periodic offset consumes substantial energy, reducing the energy conversion rate. If the floating rotor deflection is to be eliminated, it must be ensured that the airflow from both nozzles is blowing simultaneously toward the circular surface or the notch surface. The three-notch rotor cannot eliminate the offset due to its structure. When the number of notches is even, the rotor has a centrosymmetric structure, as shown in Figure 7. In both the four-notch rotor and the six-notch rotor, when the nozzles are at position 1, the radial component of the airflow flow can be completely canceled out because the left and right nozzles blow simultaneously onto the circular surface. When the nozzle is at position 2, both the left and right nozzles blow towards the notch surface, and the two radial directions can still cancel each other so that the radial forces on the rotor are balanced. With a centrally symmetrical rotor, both nozzles blow simultaneously onto the notch or circular surface regardless of the rotor rotation angle, eliminating rotor drift and thus achieving higher energy conversion rates, which can be considered an improved solution for airflow energy harvesters.

## 3. Simulation Analysis of Centrosymmetric Rotor

### 3.1. Analysis of the Output Performance

In the airflow energy harvester, two factors influence the magnitude of the peak voltage. One is the rotation speed ω of the floating rotor. Higher speed led to higher induction electromotive force in the coil. The other one is the energy conversion factor *ρ_ECF_*. The quotient of the peak voltage and the speed of the rotor is the value of the energy conversion factor. The peak voltage *E_P_* can, therefore, be expressed by Equation (10).

(10) $$E_{P}=\omega \rho_{ECF}\;$$

**Figure 8:**
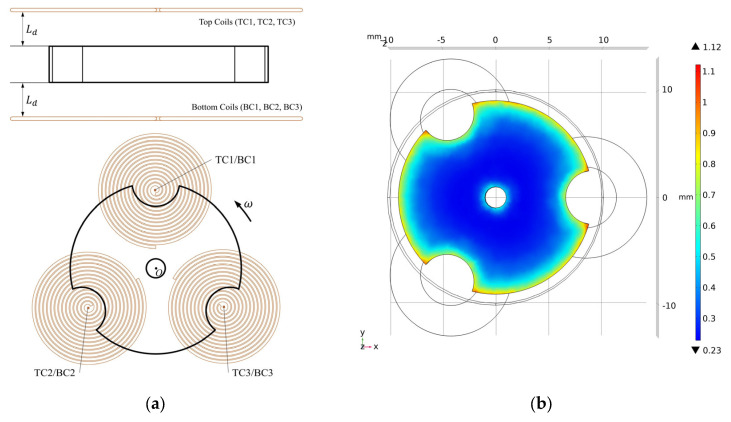
The energy conversion part: (**a**) schematic diagram of position relation of the three-notch rotor and coils; (**b**) voltage simulation model by COMSOL.

**Figure 9:**
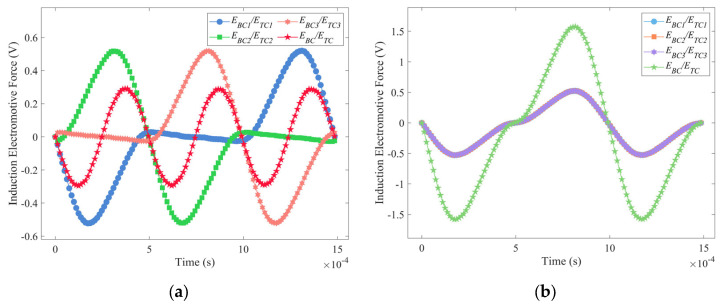

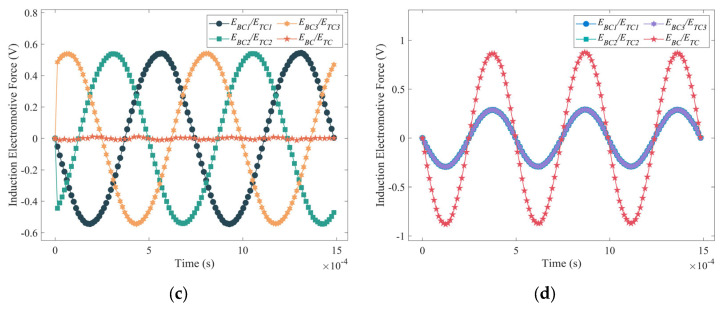
Electromotive force and total voltage of the three coils of the four rotors: (**a**) two-notch rotor; (**b**) three-notch rotor; (**c**) four-notch rotor; (**d**) six-notch rotor.

The energy conversion part of the energy harvester consists of two shaped coils made up of three circular coils connected in series, which are arranged above and below the floating rotor, as shown in Figure 8a. The simulation is modeled in COMSOL Multiphysics 5.6 as shown in Figure 8b, with the structure parameters selected from Table 1. The pushing magnet, pulling magnet, and floating magnet are made of NdFeB 52 and have a maximum magnetic energy of 400 kA/m^3^. The simulation model shown in Figure 8 is used to simulate the output voltages of two-notch, three-notch, four-notch, and six-notch rotors. The speed of three centrosymmetric rotors and the three-notch rotor is set to 20,000 rpm and the simulation time is set to the time required for half a revolution. Figure 9 shows the induction electromotive force corresponding to the four rotors, respectively.

As seen in Figure 9a,c, the phase of the induced electric potential generated by the three coils to be different, which causes the voltages in the three coils to cancel each other out, and the total voltage *E_TC_* (*E_BC_*) in the top coils (bottom coils) is less than the sum of the voltages of the three coils, as expressed in Equation (11). Moreover, the value of *E_TC_* (*E_BC_*) is always zero when using the four-notch rotor. In contrast, as seen in Figure 9b,d, the *E_TC_* (*E_BC_*) of both the three-notch and six-notch rotors are three times the electromotive force in a single coil, as expressed in Equation (12). Furthermore, the total voltage of the three-notch rotor is significantly higher than that of the six-notch rotor by a factor of approximately two. The energy conversion factors *ρ_ECFS2_*, *ρ_ECFS3_*, *ρ_ECFS4_* and *ρ_ECFS6_* of the four rotors in a single coil are calculated from Equation (10), and the values of them are 0.0260 mV/rpm, 0.0263 mV/rpm, 0.0272 mV/rpm, and 0.0146 mV/rpm, respectively. According to Equation (13), the centrosymmetric rotors may have a higher output performance. The coil arrangement of the three-notch rotor is not suitable for the centrosymmetric rotors, which results in their low total voltage.

(11) $$E_{BC}\left(E_{TC}\right)<E_{BC1}\left(E_{TC1}\right)+E_{BC2}\left(E_{TC2}\right)+E_{BC3}\left(E_{TC3}\right)$$

(12) $$E_{BC}\left(E_{TC}\right)=E_{BC1}\left(E_{TC1}\right)+E_{BC2}\left(E_{TC2}\right)+E_{BC3}\left(E_{TC3}\right)\;$$

(13) $$\rho_{ECFS4}>\rho_{ECFS3}>\rho_{ECFS2}>\rho_{ECFS6}$$

**Figure 10:**
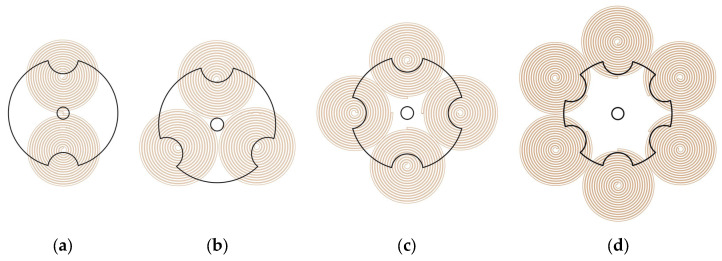
The improved coil arrangement of the centrosymmetric rotors: (**a**) two-notch rotor; (**b**) three-notch rotor; (**c**) four-notch rotor; (**d**) six-notch rotor.

**Figure 11:**
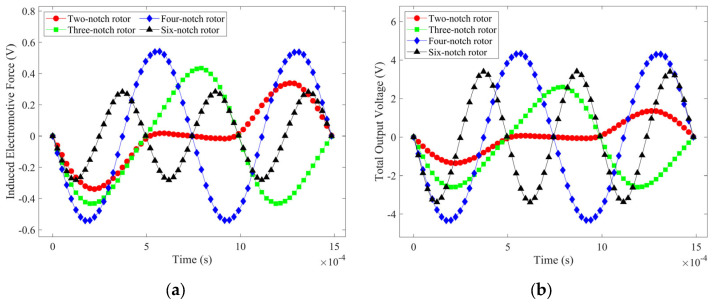
Electromotive force in a single coil and total output voltage of the four rotors with improved coil arrangements: (**a**) induction electromotive force in a single coil; (**b**) total output voltage.

With the centrosymmetric rotors, the existing coil arrangement must be changed to avoid the induction electromotive force canceling each other out. The improved coil arrangement is shown in Figure 10; several circular coils are fixed on each of the upper and lower HOPG plates that are connected in series and tangent to each other, with the number of them equal to the number of notches of the floating magnet. Using these improved coil arrangements, the induction voltages of the four rotors were simulated in COMSOL Multiphysics 5.6 and plotted in Figure 11. In Figure 11a, the peak electromotive force in the single coil *E_SC_* of the two-notch rotor, three-notch rotor, four-notch rotor, and six-notch rotor are 0.3392 V, 0.4344 V, 0.5415 V, and 0.2839 V, respectively. The output voltage *E* is calculated according to Equation (14), where *N* is the number of notches in each rotor. In Figure 11b, the peak output voltages of four rotors are 0.6784 V, 2.6064 V, 4.332 V, and 3.4068 V, respectively. Both the total output voltage and the electromotive force in the individual coils are at their maximum when using the four-notch rotor. As a result, the four-notch rotor has the highest energy conversion factor among the four rotors, boosting the output voltage by 1.7256 V compared to the three-notch rotor.

(14) $$E=E_{TC}+E_{BC}=2NE_{SC}\;$$

**Figure 12:**
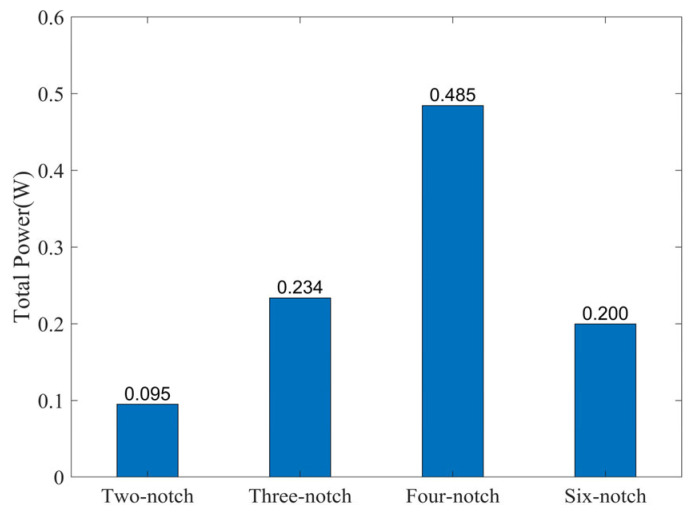
The output power of the four rotors.

The simulation gives a resistance of 2.42 Ω for a single circular coil. The power of four rotors was calculated from the total output voltage and resistance, as shown in Figure 12. The four-notch rotor has the highest output power of 485 mW. In summary, the four-notch rotor is the best in terms of both energy conversion factor and output power.

### 3.2. Analysis of Levitation Characteristics

**Figure 13:**
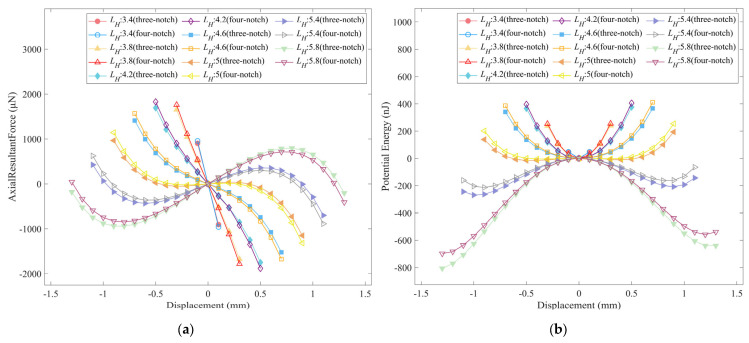
The axial resultant force and potential energy of the three-notch rotor and the four-notch rotor: (**a**) the axial resultant force; (**b**) the potential energy.

The levitation characteristics of the three-notch and four-notch rotors were calculated using a joint MATLAB and COMSOL simulation. The magnetic and diamagnetic forces of the four-notch rotor are smaller compared to the three-notch rotor due to the lower magnetic induction strength, which makes the axial combined forces and the potential energy of the two types of floating rotor follow approximately the same trend, with slight differences, as shown in Figure 13a,b. This means that there are also differences in the levitation characteristics [25] of the two rotors.

**Figure 14:**
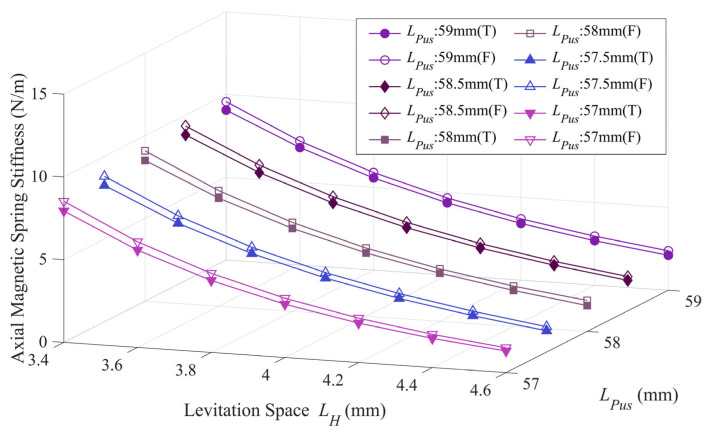
Comparison of the axial magnetic spring stiffness of the three-notch rotor and the four-notch rotor.

The axial magnetic spring stiffness of the floating rotor represents the ability of the energy harvester to cope with external axial excitation. The larger the axial spring stiffness, the higher the axial stability. As seen in Figure 14, the axial magnetic spring stiffness of the energy harvester is significantly increased when using the four-notch rotor. At *L_Pus_* values of 59 mm, 58.5 mm, 58 mm, 57.5 mm, and 57 mm, the axial magnetic spring stiffness increases by an average of 7.886%, 8.328%, 9.915%, 10.939%, and 11.263%, respectively. The overall average increase in axial magnetic spring stiffness is 9.666%.

**Figure 15:**
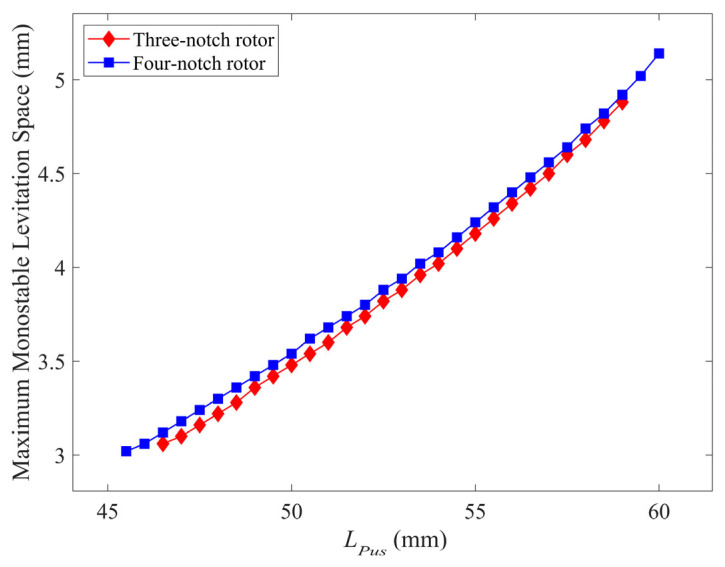
Comparison of maximum monostable levitation space at multiple levitation points for the three-notch rotor and the four-notch rotor.

In Figure 15, each value of *L_Pus_* represents a levitation point, and at each levitation point, there is a maximum monostable levitation space *L_Hmax_*. When using the four-notch rotor, *L_Pus_* takes on a larger range of values and more levitation equilibrium points can be selected. For the same value of *L_Pus_*, the maximum monostable levitation space of the four-notch rotor is somewhat enhanced, with an overall average enhancement of 1.67%. Furthermore, the maximum monostable levitation space’s maximum value is increased from 4.88 mm to 5.14 mm. The larger maximum monostable levitation space facilitates observation of the levitation state of the floating rotor and reduces the possibility of friction between the floating rotor and the coil due to axial excitation.

**Figure 16:**
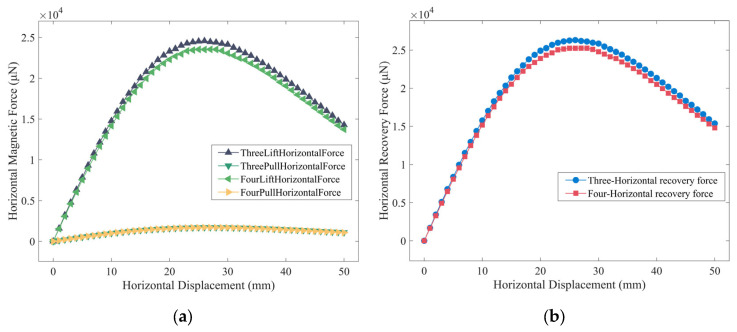
The horizontal component of magnetic force and horizontal recovery force for the three-notch rotor and the four-notch rotor: (**a**) comparison of the horizontal component of magnetic forces; (**b**) comparison of horizontal recovery forces.

As shown in Figure 16a, the horizontal magnetic force on the four-notch rotor has decreased to some extent, but the reduction is not significant, with the horizontal component of the magnetic force of the pushing magnet decreasing by an average of 4.56% and the horizontal component of the magnetic force of the pulling magnet decreasing by an average of 2.71%. In Figure 16b, the horizontal recovery force for the four-notch rotor has the same trend, with an average reduction of 3.97% compared to the three-notch rotor. The reduction in horizontal recovery force does not affect the performance of the energy harvester as the four-notch rotor does not offset during operation. Moreover, the reduction is within 5%, does not significantly impact horizontal stability, and can cope with horizontal excitation in the operating environment.

## 4. Experimental Verification

### 4.1. Levitation Characteristics Test Experiments

**Figure 17:**
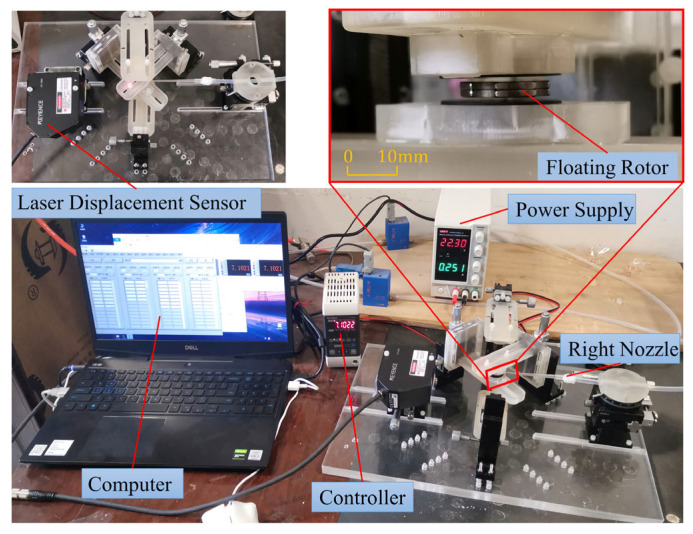
Experimental platform for the levitation characteristics of the airflow energy harvester.

The experimental platform of the improved airflow energy harvester is built according to Figure 1, as shown in Figure 17, with the same structural parameters as Table 1. The experiments used two 1.5-mm thick floating rotors stacked together in place of a 3-mm thick floating rotor. The prototype is fixed to a non-magnetic acrylic plate with the aid of multiple adjustment tables and connections printed from photosensitive resin to ensure that the pushing magnet, the pulling magnet, the upper HOPG, and the lower HOPG can be arranged coaxially. Moreover, that the adjustment tables adjust the axial distance between them. In this experiment, only the right-side nozzle was used, and the airflow was regulated by a single flow controller (MFC300). A laser displacement sensor (LK-G80) is placed on the left-hand adjustment table to measure the horizontal displacement of the floating rotor. LK-G80 is connected to the controller (LK-G3001) to display the displacement data, which are recorded by a PC connected to the controller via USB.

Experiments were first carried out on the maximum monostable levitation space at multiple levitation equilibrium points for the three-notch rotor and the four-notch rotor. The axial positions of the pushing magnet, the pushing magnet, and the two HOPG sheets are changed so that the floating rotor is at different levitation equilibrium points. The thickness of each component and the axial distance between them and the base plate are measured using Vernier calipers. The distance between the upper and lower HOPG plates was calculated from the measured data to be the maximum monostable levitation space and recorded in Table 2. As can be seen from Table 2, as *L_Pus_* decreases, the rotor can be kept levitation by reducing *L_Pul_*. When the value of *L_Pus_* is reduced from 55.365 mm to 51.365 mm, the maximum monostable levitation space *L_Hmax3_* is reduced from 4.73 mm to 3.89 mm for the three-notch rotor and *L_Hmax4_* is reduced from 4.81 mm to 3.97 mm for the four-notch rotor. Meanwhile, *L_Hmax4_* is consistently greater than *L_Hmax3_*, with an average improvement of 2.05%, in line with the simulation results in Section 3.

**Figure 18:**
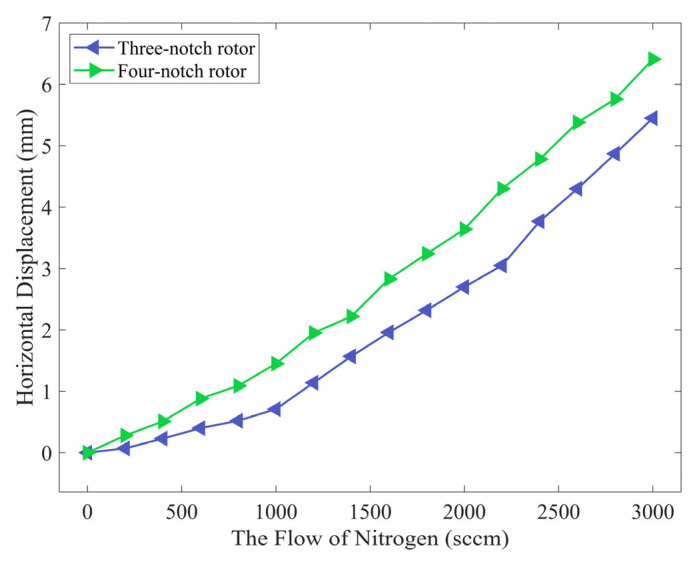
Horizontal displacement of the three-notch rotor and the four-notch rotor at different airflow rates.

Both rotors are tested for horizontal recovery force when *L_Pus_* is 55.365 mm. The airflow from the nozzle is increased from 0 sccm to 3000 sccm in 200-sccm increments. The horizontal displacement of the floating rotor is measured by a laser displacement sensor (LK-G80, Keyence); the larger the horizontal displacement value, the smaller the horizontal recovery force. The data are further processed and plotted in Figure 18.

In Figure 18, the horizontal displacement of the four-notch rotor is greater than that of the three-notch rotor at any airflow rate. The horizontal displacement reaches a maximum at an airflow rate of 3000 sccm, when the maximum horizontal displacement is 5.45 mm and 6.45 mm for the three-notch rotor and the four-notch rotor, respectively, with an increase of 17.61%. The experimental results agree with the analysis in that the horizontal recovery force of the four-notch rotor is reduced.

### 4.2. Output Performance Test Experiments

**Figure 19:**
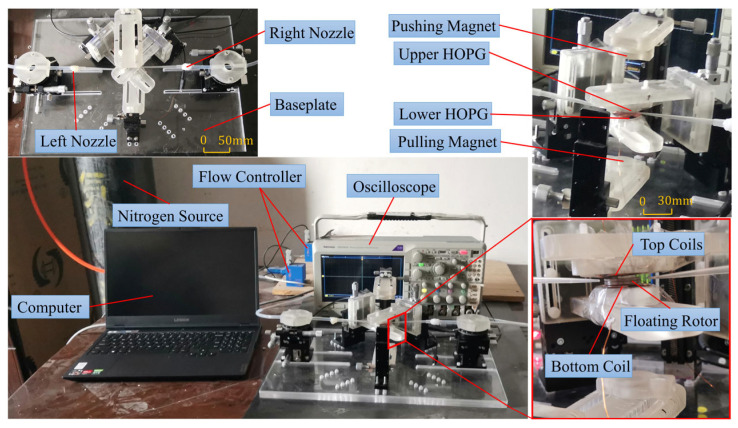
Experimental platform for the airflow energy harvester.

**Figure 20:**
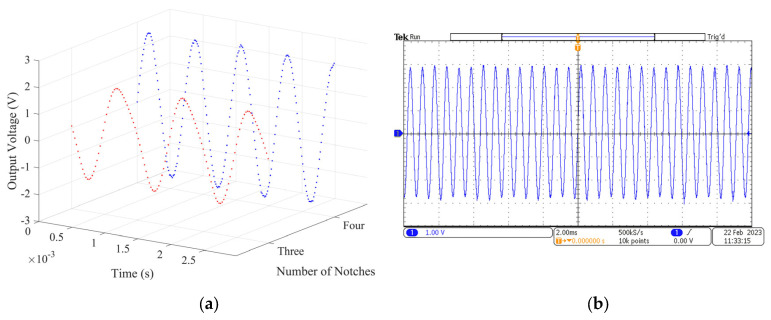
The output voltage of the three-notch rotor and the four-notch rotor: (**a**) voltage data of the two rotors; (**b**) voltage wave of the four-notch rotor.

The experimental platform for testing the output performance is shown in Figure 19. Connected to a computer, two flow controllers (MFC300) are used to regulate the airflow of the left and right nozzles. Tektronix oscilloscope is used to display the output waveform of the energy harvester. The coil arrangements of the three-notch and four-notch rotors are fixed on the upper and lower HOPG sheets in turn. The airflow rate of the two nozzles was set to 3000 sccm, and the output performance of the energy harvester prototypes with the three-notch rotor and four-notch rotor was tested. The induced voltage in the coil is generated by the varying magnetic induction strength. Each time the notch surface of the rotor passes over the coil, the voltage in the coil peaks and troughs once. This means that for one revolution of the rotor, the number of peaks and troughs in the output voltage is equal to the number of notches in the rotor. Thus, the rotational speed of the rotor can be calculated from the voltage waveform. The output voltage waves are shown in Figure 20.

In Figure 20a, the red dots represent the voltage of the three-notch rotor, the blue dots represent the voltage of the four-notch rotor. The peak voltage is 1.924 V for the three-notch rotor and 2.709 V for the four-notch rotor, with approximately 40.80% increase. The voltage waveform displayed on the oscilloscope gives a maximum speed of 20,160 rpm for the three-notch rotor and 21,367 rpm for the four-notch rotor. The rotation speed increase is about 5.99% because air resistance is increasing with rotor speed at the same time. The oscilloscope voltage wave of the four-notch rotor is shown in Figure 20b. The total resistance of the six coils connected in series was 18.9 Ω and the total resistance of the eight coils connected in series was 26.5 Ω. For the energy harvesters with three-notch rotor and four-notch rotor, the output power increased from 97.93 mW to 138.47 mW with an increase of 41.40%. Furthermore, the energy conversion factor increased from 0.095 mV/rpm to 0.127 mV/rpm with an increase of 32.68%. The sum of the kinetic energy of the airflow from the left and right nozzles per second is the input power of the experimental prototype, which is approximately 238.16 mW. The energy conversion rate of the energy harvester is equal to the ratio of the output power to the input power, which is 41.12% for the three-notch rotor and 58.14% for the four-notch rotor, with an increase of 41.39%. The four-notch rotor can output higher voltages and power while keeping the input power constant, thus significantly increasing the energy conversion rate. According to Equation (10), the output voltage of the airflow energy harvester is related to the rotation speed and the energy conversion factor. The four-notch rotor has a higher rotational speed and a higher energy conversion factor, so it can output higher power and the energy conversion rate is significantly increased.

The four-notch rotor significantly increases the power output and energy conversion rate of the energy harvester, which means that more electronic devices can be loaded and the collected wind energy can be converted into electricity more efficiently. The energy harvester can harvest the wind energy in the environment more effectively and offer workable output with a limited airflow input.

## 5. Conclusions

This paper investigates the airflow energy harvester using the push–pull diamagnetic levitation structure. Instead of using the three-notch rotor, a four-notch rotor was introduced into the energy harvester, and a corresponding comparison study was carried out to verify the improvement. The centrosymmetric rotors are required to eliminate the periodic offset. Simulations in COMSOL Multiphysics 5.6 of the output performance of multiple floating rotors, have determined that the four-notch rotor has the best output performance. After the improvement, the levitation characteristics of the four-notch rotor were changed due to its reduced magnetic induction strength and mass. 

The axial magnetic spring stiffness increased by an overall average of 9.666%, and the maximum monostable levitation space increased by an average of 1.67%. However, there is a reduction in horizontal response force of approximately 3.97%, which does not affect horizontal stability due to the elimination of the floating rotor horizontal offset. An experimental platform was set up to verify the output and levitation characteristics. According to the experimental results, when the airflow is 3000 sccm, the peak voltage of the four-notch rotor can reach 2.709 V, and the maximum speed can reach 21,360 rpm. The peak voltage, maximum rotation speed, output power, energy conversion factor, and energy conversion rate of the four-notch rotor are respectively increased by 40.80%, 5.99%, 41.40%, 32.68%, and 41.39%, compared to the three-notch rotor. At the same time, the variation in levitation characteristics is consistent with the simulation results. The use of the four-notch rotor also substantially improves output performance, particularly by increasing the energy conversion rate from 41.12% to 58.14%, while increasing the maximum monostable levitation space and axial magnetic spring stiffness, demonstrating that this is a proven improvement solution.

## Figures and Tables

**Table 1:** Parameters of the push–pull airflow energy harvester.

Parameter	Value|Material
Pushing magnet, Pulling magnet, Floating rotor	NdFeB-52
Pushing magnet	∅19 × 6.35 (mm)
Pulling magnet	∅10 × 2 (mm)
Radius of the floating rotor	9 (mm)
Thickness of the floating rotor	3 (mm)
Radius of the central bore of the floating rotor	1 (mm)
Radius of the notches of the floating rotor	2.5 (mm)
Outer diameter of circular coils	11.5 (mm)
Inner diameter of circular coils	0 (mm)
Wire diameter for circular coils	0.1 (mm)
*L_d_*	0.5 (mm)

**Table 2:** Experimental data on maximum monostable levitation space.

Number	*L_Pus_* (mm)	*L_Pul3_* (mm)	*L_Pul4_* (mm)	*L_Hmax3_* (mm)	*L_Hmax4_* (mm)
1	55.365	46.885	47.385	4.73	4.81
2	54.365	41.885	42.385	4.53	4.63
3	53.365	38.385	39.285	4.31	4.41
4	52.365	35.385	35.885	4.09	4.17
5	51.365	33.485	33.885	3.89	3.97

## Data Availability

The data presented in this study are available on request from the corresponding author.
